# Aerosol drug delivery to the lungs during nasal high flow therapy: an in vitro study

**DOI:** 10.1186/s12890-019-0807-9

**Published:** 2019-02-15

**Authors:** Martin Wallin, Patricia Tang, Rachel Yoon Kyung Chang, Mingshi Yang, Warren H. Finlay, Hak-Kim Chan

**Affiliations:** 1 Advanced Drug Delivery Group, School of Pharmacy, The University of Sydney Faculty of Medicine and Health, Sydney, NSW 2006 Australia; 2 Department of Pharmacy, University of Copenhagen, Universitetsparken 2, DK-2100 Copenhagen, Denmark; 3 Department of Mechanical Engineering, University of Alberta, Edmonton, T6G1H9 Canada

**Keywords:** Aerosol, Powders, Inhalable drugs, Nasal cannula, Pulmonary disease, chronic obstructive, Lungs, Nasal high flow

## Abstract

**Background:**

Aerosol delivery through a nasal high flow (NHF) system is attractive for clinicians as it allows for simultaneous administration of oxygen and inhalable drugs. However, delivering a fine particle fraction (FPF, particle wt. fraction < 5.0 μm) of drugs into the lungs has been very challenging, with highest value of only 8%. Here, we aim to develop an efficient nose-to-lung delivery system capable of delivering improved quantities (FPF > 16%) of dry powder aerosols to the lungs via an NHF system.

**Methods:**

We evaluated the FPF of spray-dried mannitol with leucine with a next generation impactor connected to a nasopharyngeal outlet of an adult nasal airway replica. In addition, we investigated the influence of different dispersion (20–30 L/min) and inspiratory (20–40 L/min) flow rates, on FPF.

**Results:**

We found an FPF of 32% with dispersion flow rate at 25 L/min and inspiratory flow rate at 40 L/min. The lowest FPF (21%) obtained was at the dispersion flow rate at 30 L/min and inspiratory flow rate at 30 L/min. A higher inspiratory flow rate was generally associated with a higher FPF. The nasal cannula accounted for most loss of aerosols.

**Conclusions:**

In conclusion, delivering a third of inhalable powder to the lungs is possible in vitro through an NHF system using a low dispersion airflow and a highly dispersible powder. Our results may lay the foundation for clinical evaluation of powder aerosol delivery to the lungs during NHF therapy in humans.

## Background

Long-term oxygen therapy can improve survival in patients with chronic obstructive pulmonary disease (COPD) and chronic respiratory failure [1, 2]. Nasal high-flow (NHF) therapy is a form of respiratory support used in the hospital or emergency unit [3], mainly for management of acute hypoxaemic respiratory failure [4]. NHF therapy delivers oxygen (often warm and humidified) to patients at flow rates higher than that used in traditional oxygen therapy. Warm and humidified air may eliminate the side-effects associated with conventional oxygen therapy including upper airway dryness and irritation plus mucociliary clearance interference [3, 5]. A substantial number of COPD patients suffer from exacerbations, which are defined as an acute worsening of respiratory symptoms [6]. Acute exacerbations can be treated and sometimes prevented with inhaled antibiotics, bronchodilators or corticosteroids [7–9].

Hypoxemic patients using an NHF system may benefit from combined aerosol therapy as the etiology of hypoxemia might justify the administration of aerosolized medication [10]. In vitro studies have investigated whether pressurized metered-dose inhaler (pMDI), nebulizers or dry powder inhalers (DPI) can be combined with NHF systems for simultaneous administration of oxygen and pharmaceutical aerosols [11–16]. Réminiac et al. [11, 12] found that the position of the nebulizer or pMDI in the NHF circuit is profoundly important. Placing a nebulizer before the humidification chamber resulted in 26–32% emitted dose from the nasal prongs [11], whereas placing a pMDI immediately upstream of the nasal cannula resulted in 12% emitted dose. Ari et al. [13] and Bhashyam et al. [14] performed experiments with similar nebulizer setups and achieved 2–11%, and 19–27% emitted doses, respectively. Perry and his team [15] placed a nebulizer further away from the humidification chamber (closer to the nasal prongs) and found the emission efficiency being only 2.5% in the study. Dugernier et al. [10] reported that lung deposition in vivo was 4 and 1% with a vibrating-mesh nebulizer and a jet nebulizer, respectively.

Dry powders were thought to be incompatible with an NHF system because of humidified air [17]. Water may adsorb to the surface of dry powders when the humidity is high, thereby compromising the flowability and dispersibility of the powders due to agglomeration and increased adhesiveness [18]. The use of dry powders in such systems has been neglected for that reason [16, 19, 20]. Nevertheless, we have previously shown that heated and humidified air could disperse mannitol powders as effectively as dry air [16]. However, the predicted lung dose was only 8% in that in vitro setting, limiting its clinically utility [16].

In the present study, we aimed to develop an efficient nose-to-lung delivery system using a DPI device coupled to a NHF system that can overcome the current clinical and technical limitations, with improved delivery (FPF > 15%) of powder aerosols to the lungs.

## Methods

### Materials

Mannitol was supplied from Pharmaxis Ltd. (Sydney, NSW, Australia). Tween® 80 and l-leucine were purchased from Sigma-Aldrich (Sydney, NSW, Australia). Strata C18-U (55 μm, 70 Å, 500 mg) cartridges were purchased from Phenomenex (Sydney, NSW, Australia), Sep-Pak C18 (55–105 μm, 125 Å, 200 mg) cartridges from Waters (Sydney, NSW, Australia). Methanol and deionized water (resistivity ~ 16 MΩcm at 25°C) were of analytical grade.

### Spray-dried mannitol with l-leucine

A solution of 80% mannitol and 20% l-leucine was prepared at a total solid concentration of 2 wt% in water. L-leucine in this ratio has previously been reported to aid both moisture protection and powder dispersion to enhance aerosolization performance [21, 22]. The mixture was spray-dried using a Buchi 290 spray dryer (Buchi Labortechnik AG, Flawil, Switzerland) coupled with a conventional two-fluid nozzle for atomization. The spray dryer was run at an aspiration rate of 35 m^3^/h and an atomizing airflow of 742 L/h with constant feed rate of 1.9 mL/min. An inlet temperature of 70 ^°^C was used with recorded outlet temperature of 46–49 ^°^C. The spray-dried mannitol/leucine powder (Man+Leu) was stored inside a relative humidity controlled chamber (RH < 10%) at room temperature prior to use.

### Development of the Handihaler chamber

**Fig. 1:**
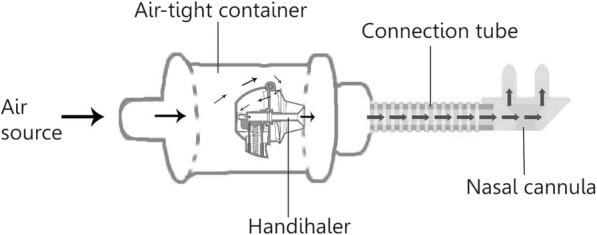
Drawing of the Handihaler chamber. Arrows indicate airflow pathway through the device. Compressed air was connected to the inlet of the container. The outlet of the chamber is connected to the nasal cannula via a connection tube. The mouthpiece of the Handihaler is inserted into a silicon adapter in the outlet of the chamber to ensure that the Handihaler is in a fixed position and that the air goes through it

We constructed the device with a Handihaler™ (Boehringer Ingelheim, Ingelheim am Rhein, Germany) in a custom-made air-tight container (Fig. 1). The experimental setup was an improvement from a previous construction by Okuda et al. [16]. The Handihaler™ is a high-resistance device, which allows powder dispersion at a much lower air flow rate compared with low-resistance devices, such as the Osmohaler™ used in our previous study [16]. The outlet of the air-tight container was connected to a large-sized nasal cannula (Optiflow™ nasal cannula, Fisher&Perkel Healthcare, Auckland, NZ) with a connection tube. The connection tube was one-quarter inch long as specified previously [16]. We used compressed air, provided by the main compressor in the building of University of Sydney, as the air source for the experiments. The flow was controlled by a valve shown Fig. 3.

### Nasal airway replica

**Fig. 2:**
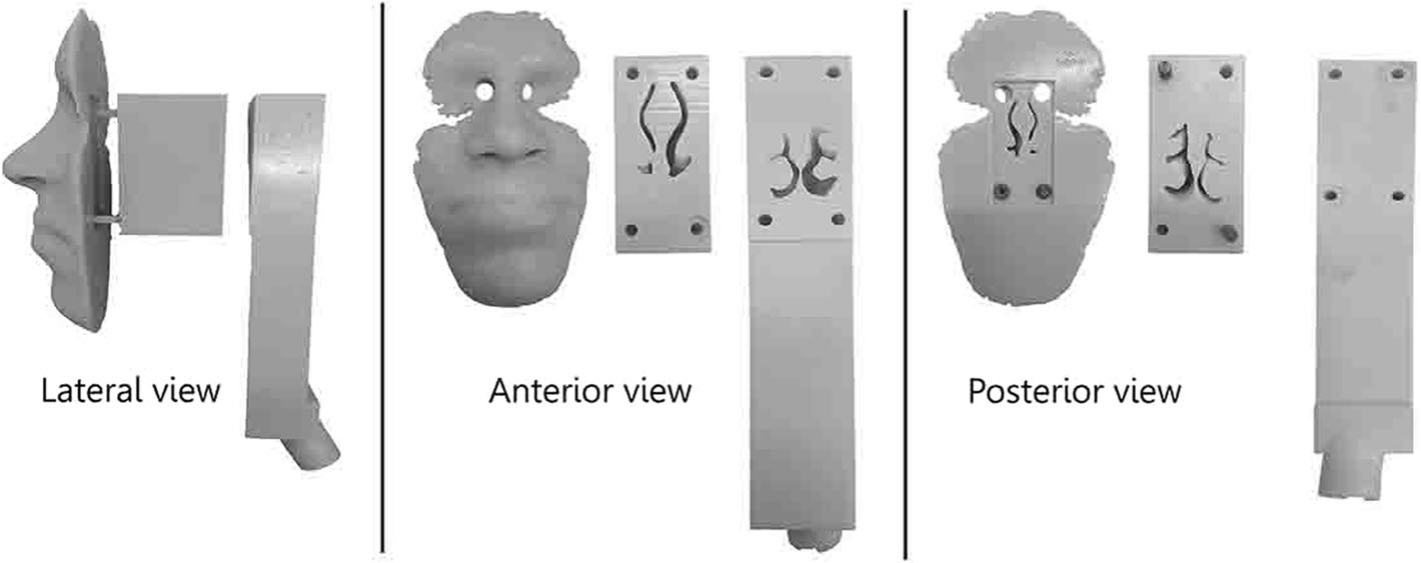
Pictures of the replica parts from three different views: Lateral, anterior and posterior. The front part (the face), mid-part and back part show the nasal vestibule, nasal turbinates, and nasopharynx, respectively

A realistic nasal airway replica (replica) was built by a fused deposition modeling 3D printing machine (PolyJet 3D, Objet Eden 350 V High Resolution 3D Printer, Stratasys Ltd., Eden Prairie, U.S.A.). The model was based on the nasal airway geometry of ‘subject 9’ of Golshahi et al. [23] obtained by magnetic resonance imaging. The volume, surface area and path length of the replica were 45,267 mm^3^, 25,086 mm^2^ and 239 mm, respectively. The material was made from acrylonitrile butadiene styrene plastic. The replica consisted of three induvial parts as shown in Fig. 2.

The interior of the replica parts was coated with 10% (*v*/v) Tween® 80 in deionized water before every experiment. Tween® 80 is a non-ionic surfactant used for neutralizing the electrostatic charge of the replica surface. The coating also helps to minimize particle bounce and re-entrainment [24, 25]. Okuda et al. [16] confirmed with an electrostatic voltmeter (Isoprobe® model 244, Monroe Electronics Inc., New York, U.S.A.) that 10% (v/v) Tween® 80 neutralizes the electrostatic charge. The replica parts were left to dry for one hour in a closed perspex box. The box was heated to 37–42°C to make the solvent evaporate faster. The dry parts were assembled with nuts and bolts. Finally, we sealed all junctions with Blu Tack (Officeworks, Sydney, Australia).

### Particle size distribution and powder emission from Handihaler

We measured the particle size distribution (PSD) of the spray dried powders by laser diffraction (Spraytec®, Malvern Instruments, Worcestershire, UK). The measurements were conducted in ambient conditions (23 ± 1°C, 50 ± 5% RH). We determined the powder emission using compressed air at dispersion flow rates (DFR) 20, 25, and 30 L/min. The flow rates were selected as they are within the normal range for NHF therapy [26]. The airflow was adjusted with a flowmeter (TSI Inc., Model 4040, Shoreview, MN, USA). We investigated the powder emission after 4, 8 and 16 s for each DFR. A timer controlled the length of each dispersion. Forty milligrams of powder was loaded into a size three hydroxypropyl methylcellulose capsule (Vcaps®, Capsugel Australia Pty. Ltd., West Ryde, Australia). We weighed the capsule and device on an analytical balance (AX205, Mettler Toledo, Switzerland) before and after each experiment to determine the emission. A large-sized nasal cannula was connected from the ‘Handihaler chamber’ to the inlet of the inhalation cell of the Spraytec®. The outlet of the cell was connected to a vacuum pump adjusted to 30 L/min.

### Next generation impactor

We used a Next Generation Impactor (NGI, Apparatus 5, USP Test chapter < 601>, Copley, UK) to investigate particle aerodynamic size distribution. Eq. 1 was used to calculate the cut-off diameter values of each of the impactor stages for flowrates higher than 30 L/min [27].

1 $$ {D}_{50,Q}={D}_{50,60L/\mathit{\min}}\cdot {\left(\frac{60}{Q}\right)}^X $$

Where *Q* is the volumetric flow rate, *X* is an experimentally determined value, and *D*_*50,60L/min*_ is the cut-off size in a given stage at 60 L/min [27]. We used Eq. 2 to calculate cut-off size in a given stage for flow rates lower than 30 L/min [28, 29].

2 $$ {D}_{50,Q}={A}^{\ast}\cdot {\left(\frac{15}{Q}\right)}^{B^{\ast }} $$

The calculated values for flow rates 20, 30 and 40 L/min are listed in Table 1.

**Table 1:** Calculated stage cut-off diameters (μm) for NGI at 20 L/min, 30 L/min, and 40 L/min

Stage	20 L/min	Flow rate 30 L/min^a^	30 L/min^b^	40 L/min
1	13.05	11.70	14.59	10.03
2	7.61	6.40	7.90	5.51
3	4.76	3.99	4.88	3.45
4	2.84	2.30	2.78	2.01
5	1.74	1.36	1.68	1.17
6	1.11	0.83	1.06	0.70
7	0.77	0.54	0.71	0.45

^a^Numbers based on Eq. 2

^b^Numbers based on Eq. 1

Table 1 was used to determine the Fine Particle Fraction (FPF) for a given flow rate. For the flow rate of 30 L/min, regardless which equations were used, the FPF was calculated for particles collected in Stage 3–8. FPF is the fraction of loaded particles with an aerodynamic diameter (D_a_) less than 5 μm (i.e. Stages 3–8) among the delivered dose. Respirable particles have a D_a_ between 1 and 5 μm.

### In vitro aerosol deposition

**Fig. 3:**
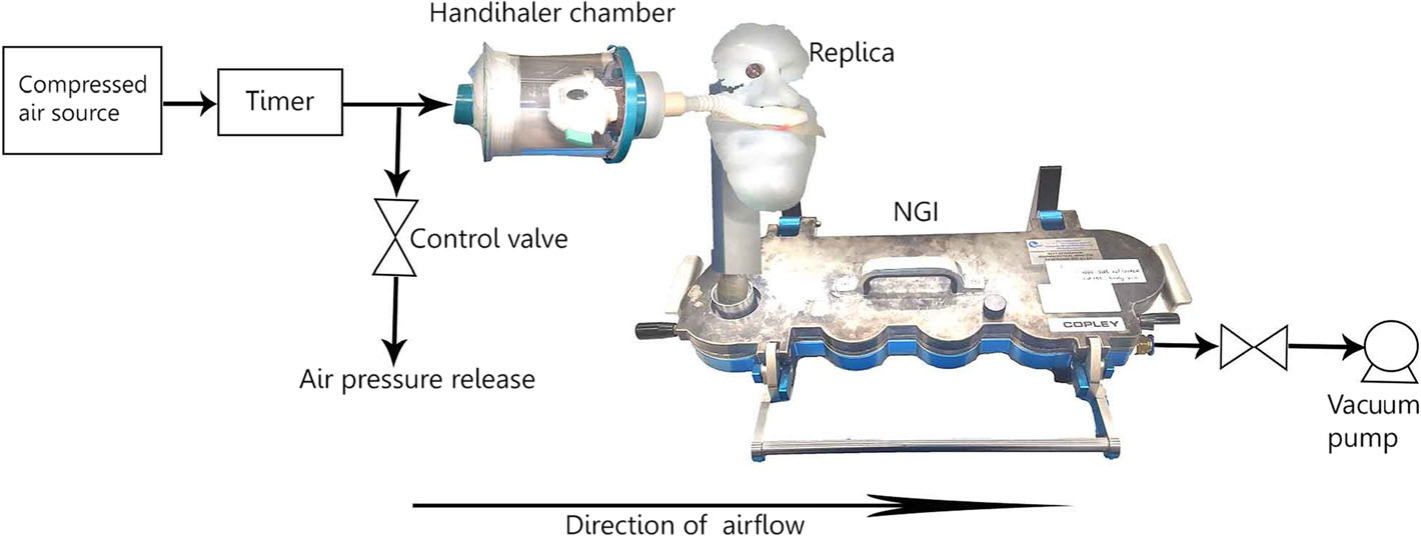
Schematic diagram of the experimental setup. Compressed air was used to aerosolize the powder from the Handihaler chamber out through the nasal cannula. A timer was used to control the length of every dispersion. A vacuum pump was used to draw powder through the replica and NGI

The schematic diagram of the experimental setup is shown in Fig. 3. The Handihaler™ was loaded with 40 ± 4 mg of powder. A large nasal cannula was inserted into the nostrils of the replica. The outlet of the replica was connected to an NGI with a vacuum pump, which generated the simulated inspiratory flow rate (IFR). Collection cups for Stages 1–8 were coated with silicone (Slipicone®, DC Products, Waverly, Australia) to minimize particle bounce and re-entrainment. The DFR and IFR were adjusted using a flowmeter (TSI Inc., Model 4040, Shoreview, MN, USA). At DFR 20 L/min, it takes at least 8 s to empty a full capsule. At 25 and 30 L/min, it takes 4 s to empty a full capsule. Long dispersions are problematic as patients cannot continuously inhale for much more than 4 s. To allow dispersions to be as short as the inspiratory phase of a person, it was split into smaller intervals. Dispersing the powder in small ‘bursts’ is more practical for actual patient use. The dispersion volume per ‘burst’ was 1 L. The ‘burst’ length was based on how fast a capsule was emptied at a given flow rate. Thus, the duration for each DFR was 3 × 3 s, 3 × 2.4 s, and 2 × 2 s, respectively (e.g. 20 L/min * 3 s = 1 L). A one-way solenoid valve with a programmable timer (RS component, Sydney, Australia) was used to control the duration of the dispersion.

The DFR and IFR were independent of each other because a patient’s breathing is independent of the air coming out of the nasal cannula. To minimize the aerosol loss in the gap between the cannula and replica, IFR was either equal to or higher than the DFR. The case of IFR being less than DFR was not considered, since a back-pressure may be created in the replica nostril, which may cause undesirable backflow of the aerosol [11, 16]. An oxygen facial mask was added to the setup to reduce losses to the ambient. A filter (Bird Healthcare, Sydney, Australia) was fitted into the mask to capture the aerosols but still allow free flow of air to avoid interfering the flow of IFR. In adults, realistic nasal airflow values are in the range of 15–40 L/min [30–32]. Since the lowest effective DFR was 20 L/min, our lowest IFR setting was set to match the value. Forty liters per minutes was the highest inspiratory flow rate.

After powder dispersion, each part of the replica was washed with deionized water to collect deposited powder. The Handihaler™, the capsule, and nasal cannula were also washed. Samples collected from the replica parts were treated with solid phase extraction (Strata® C18-U or Sep-pak® C18) to remove Tween® 80. Each cartridge was conditioned with 6 mL methanol followed by 6 mL deionized water. Five hundred microliters of the sample solution were loaded onto the cartridge. The cartridge was then washed with 500 μL of deionized water to wash the remaining mannitol off the column. After removal of Tween® 80, the samples were analyzed by HPLC.

Critical aerosol performance indices were calculated using the following equations:

$$ {\displaystyle \begin{array}{c}\% Fine\kern0.5em particle\kern0.5em fraction(FPF)=\frac{M_{<5\mu m}}{M_{load}}\cdot 100\\ {}\% Relative\kern0.5em FPF=\frac{M_{<5\mu m}}{M_{replica}+{M}_{NGI}}\cdot 100\\ {}\%\mathit{\operatorname{Re}} plica\kern0.5em deposition=\frac{M_{replica}}{M_{load}}\cdot 100\\ {}\%\mathit{\operatorname{Re}} lative\kern0.5em replica\kern0.5em deposition=\frac{M_{replica}}{M_{replica}+{M}_{NGI}}\cdot 100\\ {}\% NGI\kern0.5em deposition=\frac{M_{NGI}}{M_{load}}\cdot 100\\ {}\% Relative\kern0.5em NGI\kern0.5em deposition=\frac{M_{NGI}}{M_{replica}+{M}_{NGI}}\cdot 100\end{array}} $$

Here, M_replica_ and M_NGI_ are the mass collected in the replica and NGI, respectively. M_load_ is the loaded dose. M_<5μm_ is the mass of particles with a D_a_ < 5 μm collected from the NGI.

### HPLC quantification of mannitol

Quantification of mannitol was performed using high-performance liquid chromatography (HPLC). The Model was LC-20 (Shimadzu, Japan). The configuration used consisted of an LC-20AT pump, DGU-20A degasser, SIL-20A HT auto-sampler, RID-10A refractive index detection, CTO-20A column oven and LCSolution software. The temperature in the refractive index detector and column oven was set at 40^°^C and 85^°^C, respectively. Separation column and assay condition are shown below (Table 2)

**Table 2:** Chromatographic conditions for the experiments

Compounds	Column	Mobile phase	Flow rate, mL/min	Injection volume, μL
Mannitol	Hi-Plex Ca^2+^, 300 × 7.7 mm, 8 μm (Agilent, Sydney, Australia)	Deionized water	0.6	50

The calibration curves for mannitol were linear in the concentration range 0.05–1.1 mg/mL (r^2^ = .9999).

### Statistical analysis

We used Welch’s t-test to carry out a statistical comparison between two groups. We used a one-way analysis of variance (ANOVA) at a confidence level of 95% to identify any statistically significant differences between more than two groups. For a positive ANOVA analysis, a Tukey’s multiple comparisons test was used. A probability value (*p*-value) of less than 0.05 was considered statistically significant.

## Results

### Emission study

**Fig. 4:**
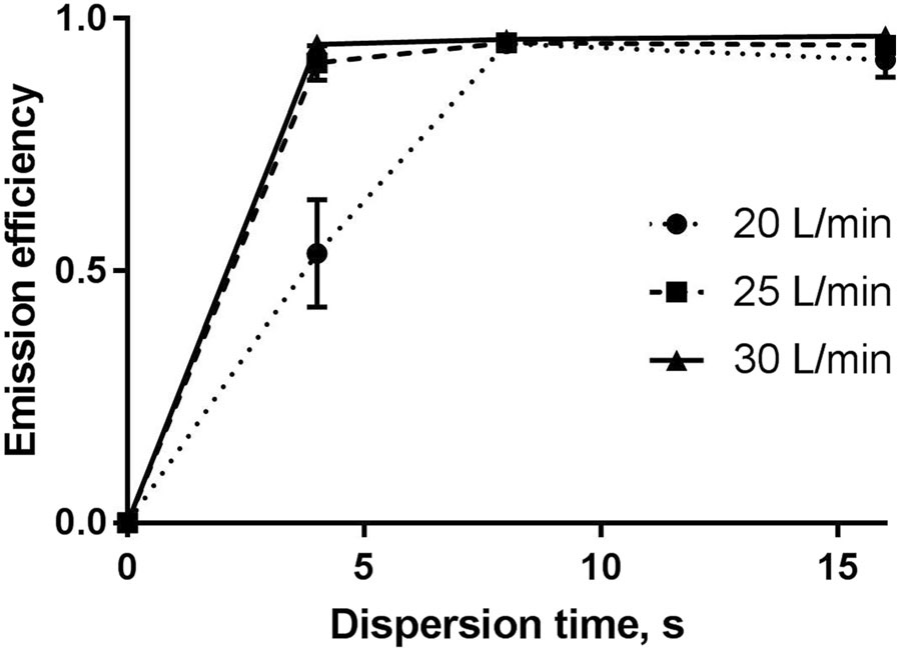
The emission efficiency of Man+Leu at different dispersion flow rates (DFR). The powder emission from the capsule was measured after 4, 8 and 16 s. The loaded dose in each experiment was 40 ± 4 mg. The nasal cannula-size was large. Each value represents the mean ± SEM (*n* = 3)

The emission efficiency of the powder is shown in Fig. 4. At DFRs of 20, 25 and 30 L/min, 53.5 ± 10.7, 91.2 ± 3.41 and 94.9 ± 0.34% of the loaded dose was emitted after 4 s, respectively. Even though 30 L/min resulted in the highest emission after 4 s, it was not significantly more efficient than 25 L/min. For the lowest flow rate, 4 s was not long enough to disperse all the loaded powder. The dispersions at 20 L/min also showed more variation. No differences were observed between the flow rates when the dispersion time was 8 s. A longer dispersion time did not further improve the emission efficiency for any DFR. We used the results to determine the length of the dispersions in the in vitro experiments.

### Particle size distribution

Particle diameters and span of the Man+Leu and mannitol aerosols exiting the nasal cannula and measured by laser diffraction are presented in Table 3. Mannitol was included in the table to show the influence of leucine. The D_50_, D_90_ and span of Man+Leu increased slightly with increasing DFR. However, these values were not significantly affected by the DFR. The D_50_ of mannitol was significantly improved by increasing the flow rate from 20 to 25 L/min. We observed no further improvement when the flow was further increased from 25 to 30 L/min. The difference between the highest and the lower flow rates was significant for mannitol as indicated in the table.

**Table 3:** Particle diameters and span of Man+Leu and mannitol emitted from a large-sized nasal cannula

DFR (L/min)	D_10_ (*μ*m)	D_50_ (*μ*m)	D_90_ (*μ*m)	Span
Man+Leu				
20	1.31 ± 0.04	3.23 ± 0.24	10.6 ± 2.11	2.86 ± 0.44
25	1.23 ± 0.03	3.28 ± 0.17	12.7 ± 2.96	3.48 ± 0.83
30	1.26 ± 0.08	3.85 ± 0.14	14.8 ± 1.48	3.51 ± 0.28
Mannitol				
20	1.95 ± 0.13	7.17 ± 1.43	62.1 ± 21.3	8.67 ± 3.29
25	1.69 ± 0.10	4.33 ± 0.33*	29.5 ± 7.03	6.36 ± 1.24
30	1.69 ± 0.08*	4.45 ± 0.35*	27.3 ± 5.63*	5.74 ± 1.04

The dose in all experiments was 40 ± 4 mg powder. Each value represents the mean ± SD (*n* = 3). D_10_, D_50_, and D_90_ are the particle diameters at 10, 50 and 90% of the cumulative particle size, respectively. Significant differences between DFR 20 and 25 L/min or 30 L/min are marked with an asterisk (* *p* < 0.05)

Clearly, Man+Leu has a more favorable PSD profile than mannitol using Handihaler™. First, the D_50_ is smaller for Man+Leu, and the span is narrower. As a result, potentially more powder can reach the lungs. Second, Man+Leu can be dispersed at a lower flow rate than mannitol. Thus, the setup has more flexibility as the powder dispersion can be achieved even at 20 L/min. However, going down to 15 L/min would result in a reduced powder emission and PSD from the Handihaler™ (data not shown).

### In vitro aerosol performance of man+Leu

Table 4 shows the in vitro aerosol performance of powder at various DFR and IFR.

**Table 4:** Aerosol performance of Man+Leu at different dispersion and inspiratory flow rates

Dispersion flow rate (L/min)	Inspiratory flow rate (L/min)	Replica deposition (% of loaded dose)	NGI deposition (% of loaded dose)	FPF (% of loaded dose)
20	20	13.28 ± 2.08	24.05 ± 0.17	23.04 ± 0.21
	30	19.09 ± 0.59	26.51 ± 0.90	25.64 ± 0.91
	40	14.59 ± 1.30	24.14 ± 1.02	23.68 ± 0.99
25	25	17.40 ± 0.78	27.76 ± 1.08	27.45 ± 0.99
	40	18.76 ± 1.91	32.32 ± 0.47	32.15 ± 0.47
30	30	15.06 ± 0.74	21.72 ± 0.74	21.03 ± 0.85
	40	20.11 ± 2.14	27.16 ± 0.79	26.60 ± 0.71

The loaded dose in all experiments was 40 ± 4 mg powder. Data are represented as the mean ± SEM (*n* = 3)

Generally, the replica deposition increased when the IFR was increased across all DFRs. The only exception was at DFR 20 L/min and IFR 40 L/min. There were no significant differences between the replica deposition results. At DFR 20 L/min, the FPFs were not significantly different from each other at different IFRs. At DFR 25 L/min, the FPF was improved when the IFR was increased from 25 to 40 L/min (*p* = .0129). For DFR 30 L/min, the FPF was significantly higher when the IFR was increased from 30 to 40 L/min (*p* = .0079).

**Fig. 5:**
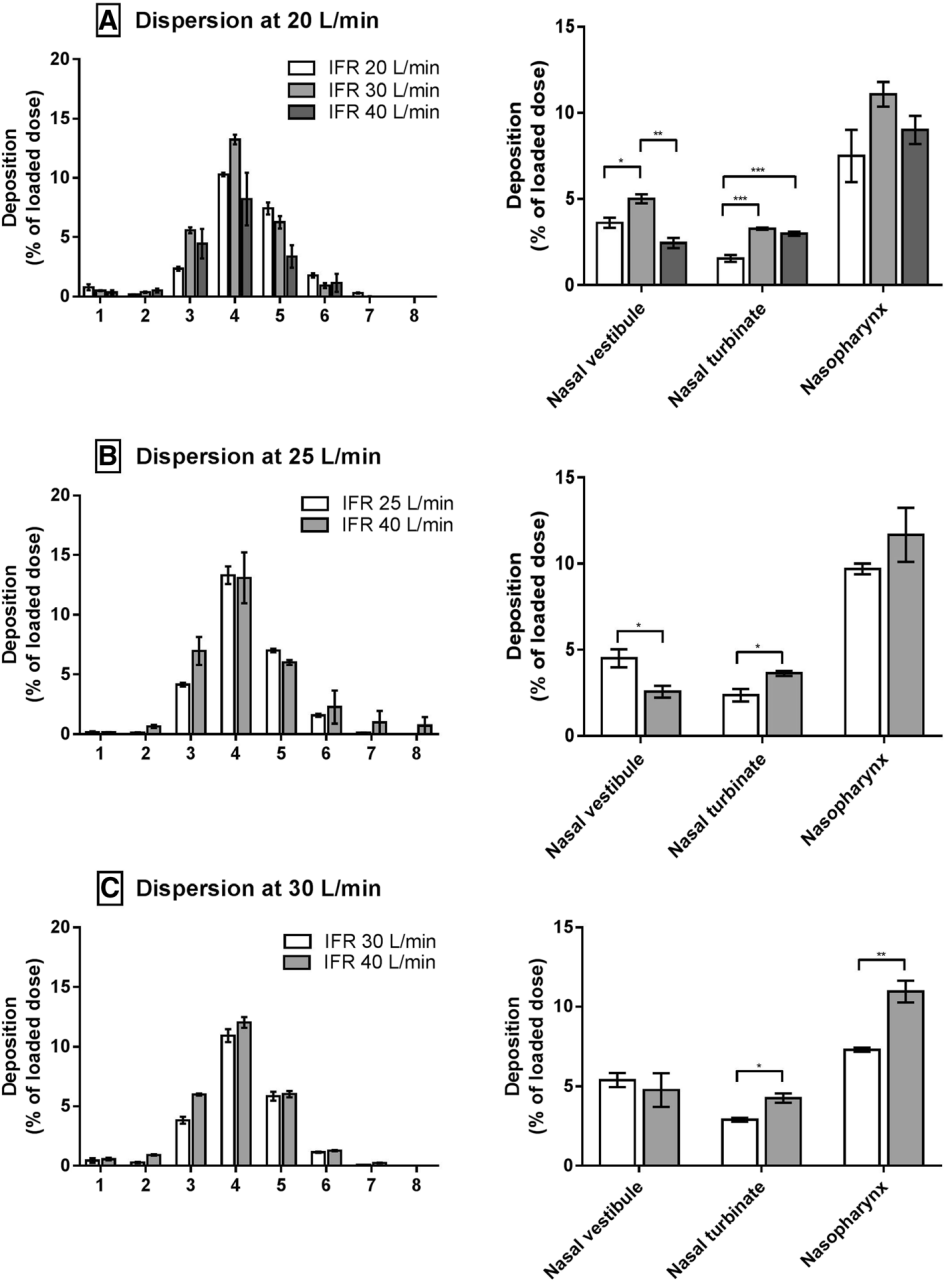
Man+Leu deposition in the NGI (Stage 1–8) and replica at various flow rate settings. A: Experiments performed at DFR 20 L/min. B: Experiments performed at DFR 25 L/min. C: Experiments performed at DFR 30 L/min. Data are presented as mean ± SEM (n = 3). Statistically significant results are marked with asterisks (*). A single asterisk indicates *p* ≤ .05. Two asterisks indicate *p* ≤ .01. Three asterisks indicate *p* ≤ .001

Because of small deposition fractions in NGI Stage 1 and Stage 2, the FPFs were similar to the NGI deposition. The deposition profiles in NGI (Fig. 5) show this clearly. In general, the distributions in the NGI were similar in all experiments. Most of the powder entering the NGI was deposited in Stage 4, irrespectively of IFR and DFR. Figure 5 also shows the deposition in different regions of the replica. It can be seen in Fig. 5 that IFR affected the deposition in the turbinates and nasopharynx.

The only exception was at DFR 20 L/min and IFR 40 L/min, which can be explained by the observations in Fig. 4. DFR 20 L/min does not always produce a consistent dispersion if the dispersion time is too short. We dispersed the powder in small bursts (3 × 3 s), so the dispersion could have been compromised. Powder deposition in the nasal vestibule was randomly distributed (Fig. 5). The aerosols leaving the cannula were mainly driven by the dispersion flow while the room air was entrained because of the inspiratory flow. The interplay between the dispersion and inspiratory flow in the area between the cannula orifice and the nostrils makes it hard to predict the deposition.

**Fig. 6:**
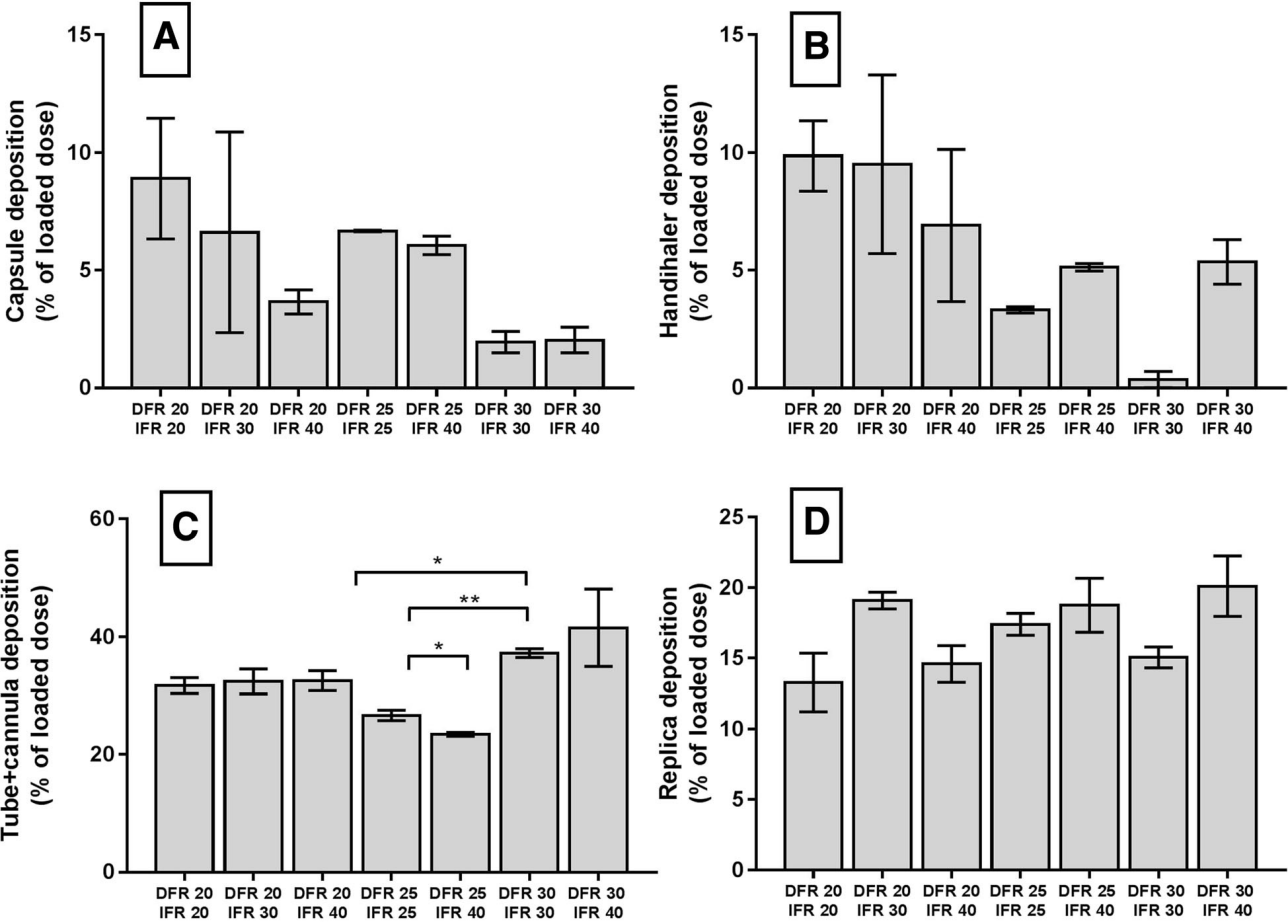
Man+Leu retention. **a**: Capsule **b**: Handihaler **c**: Cannula+connection tube **d**: Replica. Data are presented as mean ± SEM (*n* = 3). Statistically significant results are marked with asterisks (*). A single asterisk indicates *p* ≤ 0.05. Two asterisks indicate *p* ≤ 0.01

At higher DFRs, more powder was emitted from the capsule and Handihaler™, which agrees with Fig. 4. Even though we saw a trend, changing between different DFR and IFR settings did not significantly affect the retention in the Handihaler™ and capsule (Fig. 6a and b). At the same time, we found more deposition in the cannula at DFR 30 L/min (Fig. 6c). We observed the lowest retention in the cannula when the DFR was 25 L/min. IFR setting had no significant effect on the retention in the cannula. Replica deposition (Fig. 6d) is mainly affected by the inspiratory flow, especially in the turbinates and nasopharynx. The data confirm the trend (Fig. 5), but the total deposition was statistically the same across all experiments.

**Fig. 7:**
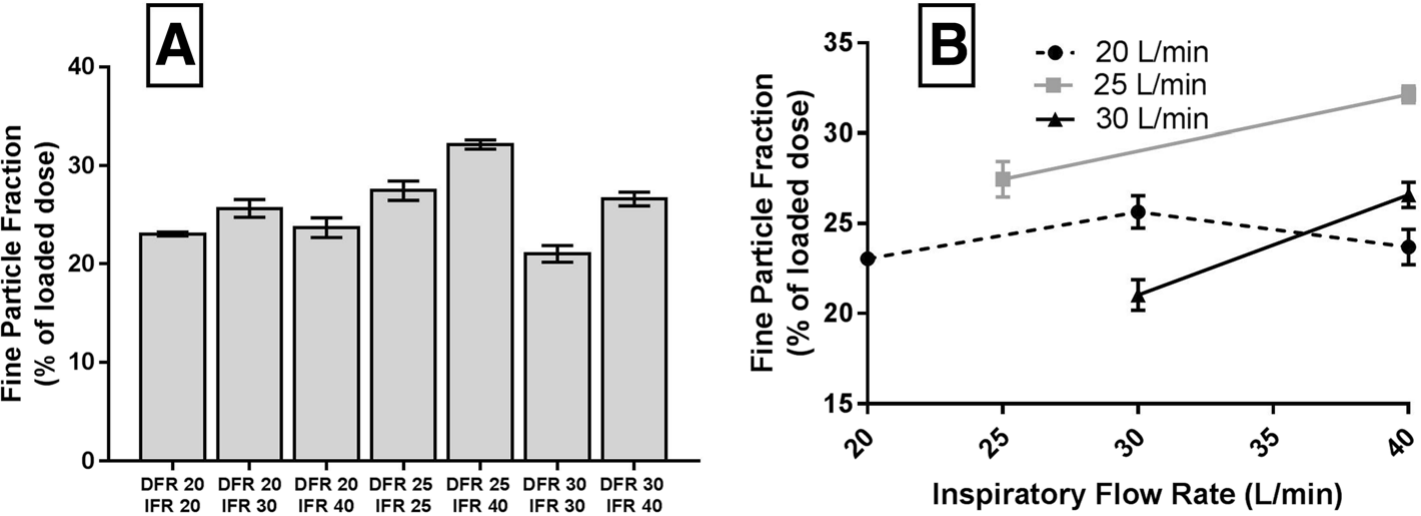
Fine particle fraction at various IFR and DFR. **a**: FPF (≤ 5.0 μm) of Man+Leu at various DFRs and IFRs. **b**: FPF (% of loaded dose) plotted against inspiratory flow rate (L/min), Data are presented as mean ± SEM (*n* = 3)

Figure 7a shows the FPF from all experiments. We obtained the biggest FPF (32.15%) at DFR 25 L/min and IFR 40 L/min. The lowest FPF (21.03%) was obtained at DFR 30 L/min and IFR 30 L/min. The FPF for all experiments was 26.24 ± 3.4%. Figure 7b illustrates how inspiratory and dispersion flow influenced the FPF. In general, FPF was increased when IFR was increased for all DFRs. As before, the only exception was at DFR 20 L/min and IFR 40 L/min, which can be explained by the observations in Fig. 4. We managed to increase the FPF by increasing the DFR from 20 L/min to 25 L/min. A higher DFR (30 L/min) did not further improve the FPF.

## Discussion

Our study demonstrated promising results compared to those in the literature [11, 13, 15, 16]. The highest FPF previously [16] was 7.99 ± 0.75% versus 32.15 ± 0.81% in the present study. A nebulizer was used in a similar setup by Reminiac and colleagues [11], where the highest respirable mass found was 10%. A pMDI was used in another study by Reminiac et al. [12], where the highest *emitted dose* was 12% during normal breathing. In other studies with nebulizers, the highest reported *emitted doses* were 27 and 11% [13, 14]. Only one in vivo study was found in the literature [10]. However, aerosol delivery to the lungs through a NHF system was only 1–4% of the nomimal dose leading the authors to conclude that the concept should be optimized further before we can expect a significant effect with nebulized antibiotics [10]. Our study may have clinical relevance, as our setup is capable of delivering high quantities of an aerosol powder to the lungs. It allows clinical evaluation of powder aerosol delivery to the lungs during NHF therapy in humans.

The high FPFs could be attributed to the relatively low DFRs. Generally, powder is dispersed more efficiently at higher dispersion flow rates [16, 33, 34]. However, higher DFRs also lead to more impaction loss [11, 16]. Therefore, if a powder were dispersible, it will be desirable to use a lower flow rate. High resistance devices can achieve efficient dispersions at a lower flow rate, e.g., the Handihaler™. A legitimate concern with a higher resistance inhaler is whether an adequate flow rate can be generated [33]. However, our setup does not rely on a patients’ ability to inhale. Instead, the Handihaler™ is activated by an external air source. Second, the good dispersibility of our Man+Leu formulation was an essential reason. The addition of 20% leucine improved the flowability and dispersibility of the powder compared with that of pure mannitol (Table 3). The D_50_ and span were smaller for Man+Leu than mannitol. The PSD values of Man+Leu were essentially the same irrespectively of the DFR. In contrast, mannitol required a higher flow rate to achieve a more satisfactory PSD, which is also what we observed previously [16].

**Fig. 8:**
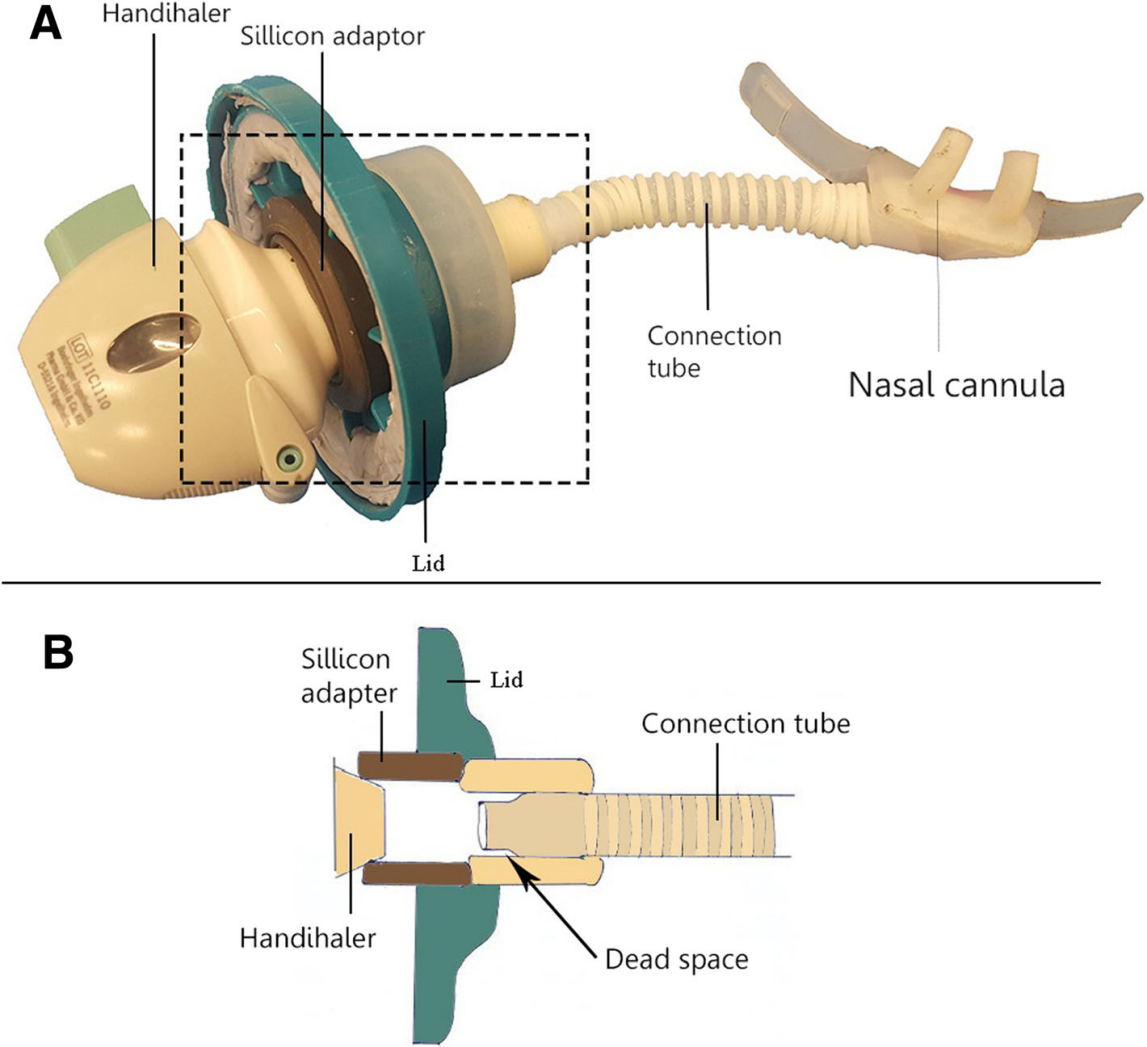
Powder loss inside the Handihaler chamber. **a**: picture of the Handihaler connected to the nasal cannula via the connection tube. The Handihaler was inserted into a silicon adapter. **b**: represents a cross sectional drawing of the dashed square

The nasal cannula and connection tube (Fig. 6c) accounted for substantial deposition loss ranging from 23.46 ± 0.59% up to 41.54 ± 11.42%. The geometry inside this region presumably caused the large deposition (Fig. 8). It can be appreciated that the inside of the Handihaler chamber connection region has flow constrictions where powder can deposit. Likewise, aerosols could be trapped in possible recirculating regions around the entry of the connection tube. Deposition could also occur in the tube. At DFR of 20, 25, and 30 L/min, Reynolds number (*Re*) values were approximately 2872, 3591 and 4309, respectively. *Re* was calculated with Reynolds equation $\left(\mathit{\operatorname{Re}}=\frac{\rho Vd}{\eta}\right)$ [35]. Thus, at 20 and 25 L/min, *Re* was in the transitional region for a circular pipe, while 30 L/min, the flow would be turbulent, although the velocity profile is likely not fully developed due to the relatively short length of the connection tube. Regardless, it is likely that turbulent dispersion likely plays some role in wall deposition in the connection tube. When the aerosols exit the connection tube and enter the nasal cannula, the flow direction changes 90 degrees. A sudden change in direction may also cause impaction in the back of the cannula, especially for particles with greater inertia. The Stokes number (Stk) is valuable to predict whether aerosols are likely to deposit in the cannula bend. According to theory, particles with Stoke number much less than one (Stk < < 1) are expected to follow gas streamlines. When Stk > > 1, particles will continue its original direction when the gas turns, rather than following the flow streamlines [35]. Using the D_50_-value of Man+Leu and a flow rate of 30 L/min, Stk = .05 which is probably small enough that particles are not much affected by changes in airflow direction. However, using the D_90_-value we found Stk = .7, indicating particles of that size probably would be affected when the airflow changes direction, which may explain our recovery of a significant amount of powder in the cannula.

We found the overall replica deposition was unaffected by the dispersion flow rate (Table 4). In contrast, it has been reported that replica deposition is expected to increase with increasing DFR [11, 16]. One possible explanation is the relatively small PSD and good dispersability of the present Man+Leu formulation. Just like the situation in the nasal cannula, small particles will follow the flow irrespective of the airflow. This indicates that the particle size predominantly determines the replica deposition. This is supported by a computational fluid dynamic deposition study, where regional deposition of nasal sprays in the airways of the nose was explored across different physical parameters [36]. The percentage of particles reaching the lungs was found to be relatively insensitive to the injection velocity whereas particle size showed a bigger influence on the deposition in the nose [36]. However, we did observe increased deposition in the nasal turbinates and nasopharynx when the IFR was increased (Fig. 7). We observed the same previously [16]. This may be due to some combination of enhanced impaction and turbulent deposition at the higher flow rates. Reynolds number *Re* in the replica can be calculated using the modified equation provided by Golshahi et al. [23]. At IFR 20, 25, 30 and 40 L/min, the replica specific *Re* were 2087, 2609, 3131 and 4174, respectively. Abrupt local diameter changes in the nasal cavity can trigger the onset of turbulence. (If air flows through a diverging duct, then the transition from laminar to turbulent can happen at a *Re* considerably lower than 2000 [37]). Thus, the onset of local turbulence or increases in separated flow region size could have caused more deposition in the replica at the higher flow rates. In addition, Stokes number Stk increases with flow rate, which may result in increased impaction.

IFR had a positive effect on the FPF in the experiments (Fig. 7b). At higher IFR more powder was drawn into the NGI. More powder in the NGI lead to a higher FPF. We had similar observations previously [16].

Our work has limitations that need to be addressed and improved for future studies. First, the current setup was not integrated into a clinically-approved NHF system. The AIRVO was not compatible with our setup. The AIRVO system could not reach a specific airflow quickly enough to be used for the short ‘burst’ in our experiments. Second, the air source was dry and not humidified oxygen. Conceptually, it would have been more accurate to use humid air instead of dry air. However, Okuda et al. [16] found the dispersibility of spray-dried mannitol was not affected by the air source due to the low exposure time. For Man+Leu powder, the effect of humidified air would probably be negligible due to the presence of leucine on the surface of the particles. Li et al. [21] found l-leucine protects powders from moisture-induced deterioration. Even if we used humidified air, the powder would only have been exposed for a short time. Additionally, the viscosity of air and oxygen do not differ much (both kinematic and dynamic viscosity). Thus, using air and not oxygen is not likely to have altered our results much since neither *Re* or Stk was much affected. Third, our design can be significantly improved. The dead space volume can be reduced by reducing the volume of the ‘Handihaler Chamber’. The silicon adapter inside the Handihaler chamber was not a perfect fit for the Handihaler™. If the Handihaler is not firmly inserted, air might bypass the device and ruin the dispersion. The connection tube should be replaced with a type with a smooth, rather than corrugated, inner surface. The Optiflow™ used here comes with a spiral corrugated connection tube. It has a relatively rough surface that could cause additional deposition. Fourth, in vitro studies with replicas of nasal airways have limitations. The extrathoracic geometries vary significantly between individuals [38]. Our replica was based on the MRI data of a single human being [23]. Finally, a vacuum pump was used during the experiment to simulate inspiration of a person. The flow was constant and is not realistic. A more realistic inspiratory airflow would use e.g. a sine function vs time. By replacing the constant flow condition with realistic breathing profiles, more representative results can be obtained. Although this study has limitations, our results demonstrate that the system can effectively deliver aerosols to the lungs.

Particle deposition in the nose and extrathoracic area is affected by the size of the airway [23, 38, 39]. Therefore, it would be valuable to validate our findings in healthy human subjects. Investigating subject-specific deposition in humans would be relevant as well. The topography of the nasal airway can be accurately determined with an acoustic rhinometer [40]. Furthermore, in vivo studies can be used to validate in vitro models. Results obtained from people with a Caucasian background may not apply to people with an Asian background as the nasal geometry is different [41]. Thus, investigating potential differences in deposition between human beings with different race would also be an entirely new topic to consider.

## Conclusion

In conclusion, we have successfully developed an in vitro physical model capable of delivering large quantities of aerosols to the lungs with a nasal cannula. The highest fine particle fraction obtained was 32%, and the lowest fraction was 21%. Our work demonstrates that dry powder inhalers may be practical for NHF systems. Our results may lay the foundation for clinical evaluation of powder aerosol delivery to the lungs during NHF therapy in humans.

## Abbreviations

ANOVA: One-way analysis of variance
COPD: Chronic obstructive pulmonary disease
DFR: Dispersion flow rate
DPI: Dry powder inhaler
FPF: Fine particle fraction
HPLC: High-performance liquid chromatography
IFR: Inspiratory flow rate
Man+Leu: Mannitol+Leucine
MRI: Magnetic resonance imaging
NGI: Next generation impactor
NHF: Nasal high flow
PSD: Particle size distribution
SD: Standard deviation; SEM: Standard error of the mean
